# Activation of EZH2 Promoter Is Mediated by MAPK Signaling Regulated Transcription Factors in Anaplastic Thyroid Cancer

**DOI:** 10.3390/ijms27146402

**Published:** 2026-07-18

**Authors:** Marcella Maringolo Cristovão, Diego Claro de Mello, Edna Teruko Kimura, Cesar Seigi Fuziwara

**Affiliations:** 1 Department of Cell and Developmental Biology, Institute of Biomedical Sciences, University of São Paulo (USP), São Paulo 05508-000, SP, Brazil; 2 Department of Biochemistry, Escola Paulista de Medicina, Universidade Federal de São Paulo (UNIFESP), São Paulo 04044-020, SP, Brazil

**Keywords:** anaplastic thyroid cancer, EZH2, MAPK signaling, promoter region, transcription factors, transcriptional control

## Abstract

Anaplastic thyroid carcinoma (ATC) is the most aggressive thyroid cancer type harboring TP53, TERT promoter and MAPK signaling alterations. Additionally, EZH2 overexpression leads to epigenetic silencing of tumor suppressor and cell differentiation genes. Here, we investigate the mechanism of EZH2 transcriptional activation in ATC. We used several luciferase reporter constructs with deletion of transcription factor (TF) binding sites identified in silico to determine EZH2 minimal promoter in ATC. MAPK signaling blockage with U0126, TF overexpression and knock-down strategies were used to evaluate EZH2 expression and reporter plasmid response, and the cross talk with TFs. As a result, we observed that EZH2 transcription is regulated by a minimal promoter of 107 bp (E3/4 region), that contains binding sites for TF NFYA, YY1 and FOXM1, highly expressed in ATC, that when deleted reduced EZH2 promoter activation. MAPK blockage reduced EZH2 and influenced YY1 and FOXM1 TF levels, while overexpression of NFYA, YY1 and FOXM1 resulted in pro-tumoral effects and EZH2 upregulation in papillary thyroid cancer cells. On the other hand, FOXM1 knock-down reduced EZH2 activation in ATC cells. Thus, we identified the minimal promoter region essential for EZH2 activation in ATC that is controlled by MAPK signaling in crosstalk with TFs.

## 1. Introduction

Thyroid cancer is the most prevalent form of endocrine malignancy [1]. About 95% of cases are classified as well-differentiated cancers, including papillary thyroid cancer (PTC) and follicular thyroid cancer, with overall good prognosis [2]. Conversely, anaplastic thyroid cancer (ATC) is rare but very aggressive, representing only 1% to 2% of all thyroid cancer cases [3], but comprises the majority of thyroid cancer-related deaths (20 to 50%) [4]. ATC is also classified as undifferentiated thyroid cancer as it lacks the expression of genes responsible for maintaining cell differentiation and the ability to uptake iodine from bloodstream, such as Sodium Iodide Symporter (NIS), resulting in refractoriness to radioiodine therapy, the primary treatment for thyroid cancers [5]. ATC progression is rapid, with an overall survival shorter than 6 months for patients [6], largely due to rapid local tumor growth and metastases induced by epithelial–mesenchymal transition (EMT).

The genetic analysis of differentiated thyroid cancers shows prevalence of alterations in the MAPK pathway present in more than 89% of PTC, driven mainly by BRAF^V600E^ (59%), mutations in RAS isoforms (13%) and fusion mutations such as RET/PTC (13%) [7]. On the other hand, ATC shows prevalence of loss-of-function-mutations in the *TP53* [8], somatic mutations in the *TERT* promoter [9], and mutations in the MAPK pathway, such as BRAF^V600E^ and RAS [10], data reinforced by the high-throughput analysis in TCGA database [11]. Recent studies have shown that combined treatment of Dabrafenib, a BRAF inhibitor, and Trametinib, an MEK1/2 inhibitor, can efficiently block the MAPK pathway in ATC. Patients with BRAF mutant ATC respond to double blockage of MAPK pathway in 56% of cases, which contributes to an overall increase in the ATC patient survival rate [12,13], although ATC still remains incurable.

Additional genetic alterations, such as deregulation in chromatin accessibility, cooperates with the main genetic drivers to promote cancer progression. In the epigenetic context, ATC can harbor inactivating mutations in the subunits of SWI/SNF complex [14], which is responsible for nucleosome remodeling to promote chromatin accessibility. This effect is opposed by the Polycomb Repressive Complex 2 (PRC2)/EZH2 that induces chromatin compaction.

Indeed, EZH2 deregulation has been reported in several types of cancer, such as melanoma, lung cancer, pancreatic, breast, prostate and thyroid cancer [15,16,17,18,19,20]. EZH2 is the methyltransferase subunit of the PRC2 complex, that promotes histone H3 trimethylation at lysine 27 (H3K27Me3), which leads to heterochromatin formation and gene silencing via cooperation with the PRC1 complex [21]. In ATC, overexpression of EZH2 contributes to repress genes related to thyroid differentiation [22,23], such as *NIS*, *TG*, and *TPO*, related to iodine metabolism, as well as the transcription factors (TFs) *PAX8* and *NKX2-1*. Moreover, we showed that blocking EZH2 induces thyroid differentiation and iodine trapping in ATC, as well as reduces tumor aggressiveness in vitro and in vivo [15]. Thus, uncovering the mechanism for *EZH2* activation in ATC is essential to understand the biology of this cancer.

In this study, we investigated the molecular mechanism for *EZH2* promoter activation in ATC and identified the minimal promoter region that majorly controls its transcription. This promoter is responsive to MAPK signaling and to TFs, such as NFYA and FOXM1, which are also regulated by MAPK signaling, indicating a regulatory network that drives *EZH2* activation in ATC.

## 2. Results

### 2.1. EZH2 Promoter Region Is Activated in Thyroid Cancer Cell Lines

**Figure 1:**
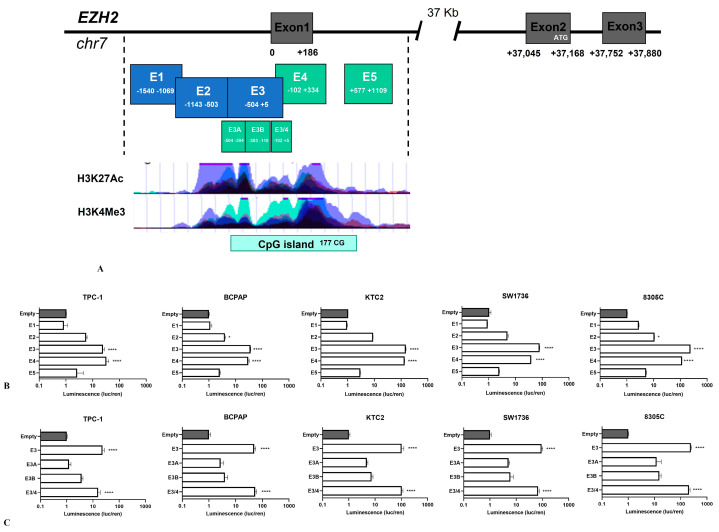
Analysis of the human *EZH2* gene promoter. (**A**): In silico analysis in the Genome Browser showed high levels of H3K4Me3 and H3K27Ac deposition around *EZH2* exon1 indicating a transcriptionally active promoter (intensity of blue color in the peaks). This region was divided into eight segments (E1, E2, E3, E3A, E3B, E3/4, E4, E5) and cloned into luciferase reporter plasmid. (**B**): Luciferase reporter assay analysis of the five longer fragments (E1, E2, E3, E4 and E5) showed higher luminescence levels for E3 and E4 fragments in all cell lines tested. (**C**): Luciferase reporter assay of three smaller fragments (E3A, E3B and E3/4) showed higher luminescence induction for E3/4, similar to E3 in all cell lines. Luciferase activity was normalized using Renilla activity. Data represented as mean ± SD (*n* = 3). Experiment performed in triplicate at least two times. * *p* < 0.05; **** *p* < 0.0001 vs. empty control.

To investigate the molecular mechanism for *EZH2* gene promoter activation in ATC, we retrieved from the Genome Browser data about the histone methylation/acetylation levels (H3K4Me3/H3K27ac) which indicates chromatin inaccessibility/accessibility. We found that the human *EZH2* gene shows strong deposition of these epigenetic markers, especially acetylation levels, associated with active promoters around *EZH2* first exon, which contains a CpG island (Figure 1A). Thus, we focused the promoter region analysis around *EZH2* exon1 by dividing this region into several smaller promoter fragments that were cloned in luciferase reporter plasmids (E1 to E5 fragments).

We observed that E3 and E4 fragments showed the highest luciferase activity compared to the empty control in all the cell lines tested, while the regions E1, E2 and E5 presented a luciferase activity that is similar to the empty control level (Figure 1B). We then performed a luciferase assay using smaller fragments derived from E3 and E4, and we observed that E3/4 presented the highest activity compared to the control, while E3A and E3B showed lower activity. The minimal promoter region E3/4 induced a strong luciferase luminescence, which is comparable to E3 alone in all cell lines (Figure 1C).

### 2.2. Transcription Factors That Regulate EZH2 Transcription Are Overexpressed in ATC

We identified the *EZH2* minimal promoter E3/4 as a fragment of 107 bp located in the −102 to +5 of TSS and used the LASAGNA algorithm to search for potential transcription factors (TFs) that may bind to E3/4 region and control its activity. We observed that E3/4 region contains binding sites for several cancer-related TFs (Table S3).

**Figure 2:**
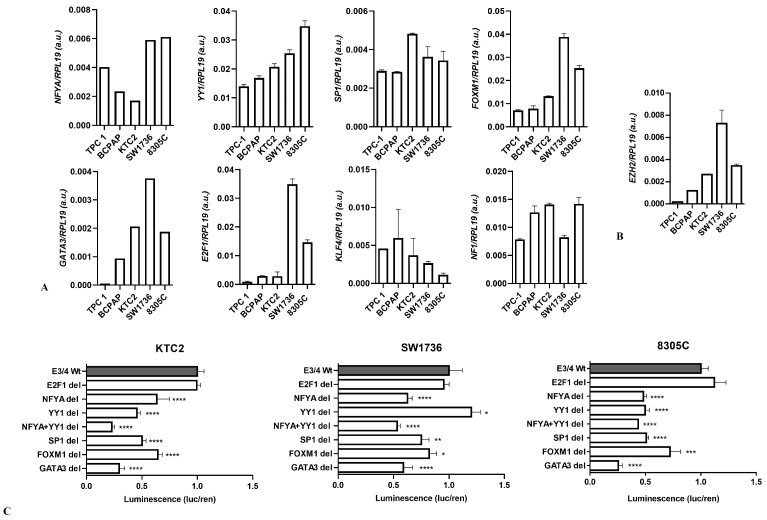
Gene expression analysis of TFs that potentially bind to *EZH2* promoter in thyroid cancer cells. *NFYA*, *YY1*, *SP1*, *FOXM1*, *GATA3* and *E2F1* show higher expression in ATC cells by qPCR (**A**) and correlate with *EZH2* expression in ATC (**B**). The gene expression was normalized using *RPL19*. (**C**): Site-directed deletion of NFYA, YY1, SP1, FOXM1 and GATA3 binding sites reduced luminescence of the E3/4 luciferase reporter plasmid. Combined deletion for NFYA + YY1 sites resulted in an additive effect in the reduction in E3/4 activation. Luciferase activity was normalized using Renilla activity. Data represented as mean ± SD (*n* = 3). Experiment performed in triplicate at least two times. a.u., arbitrary unit. * *p* < 0.05; ** *p* < 0.01; *** *p* < 0.001; **** *p* < 0.0001 vs. E3/4 Wt.

We then quantified TF expression levels in thyroid cancer cell lines and observed correlation with *EZH2* expression in ATC cells. *NFYA*, *E2F1* and *FOXM1* are highly expressed in the SW1736 and the 8305C cell lines, while *YY1*, *SP1* and *GATA3* are highly expressed in all ATC lines studied. On the other hand, *NF1* shows higher expression in KTC2 and 8305C cell lines (Figure 2A). This expression pattern correlates with *EZH2* expression levels in ATC, as we observed an overexpression in all three ATC cell lines (Figure 2B). Data retrieved from the GEPIA2 database supports the correlation between *EZH2* expression and *NFYA*, *YY1*, *SP1*, *FOXM1* and *E2F1* in the set of THCA with 521 patients with thyroid cancer and 337 control patients extracted from TCGA. Moreover, survival analysis shows that high expression of *EZH2*, *SP1*, *FOXM1* and *E2F1* correlates with a poorer prognosis in thyroid cancer patients’ survival analysis (Figure S1).

Next, we created site-directed deletions into the E3/4 region to investigate if the alteration in the predicted binding sites for the TFs analyzed would modulate *EZH2* promoter activity in ATC cells. As result (Figure 2C), for the KTC2 cell line, we observed that, in general, a reduction of 40%, 50%, 80%, 50%, 35% and 60% for deletions in the NFYA, YY1, NFYA + YY1, SP1, FOXM1 and GATA3, respectively. For the SW1736 cell line, we observed a general reduction of ~40%, 50%, 30%, 20% and 40% for deletions in NFYA, NFYA + YY1, SP1, FOXM1 and GATA3 sites, respectively. For 8305C cell line, the reductions observed were about 50%, 50%, 57%, 50%, 30% and ~75% for deletions of NFYA, YY1, NFYA + YY1, SP1, FOXM1 and GATA3 sites, respectively.

### 2.3. MAPK Signaling Inhibition Reduces EZH2 Activation

**Figure 3:**
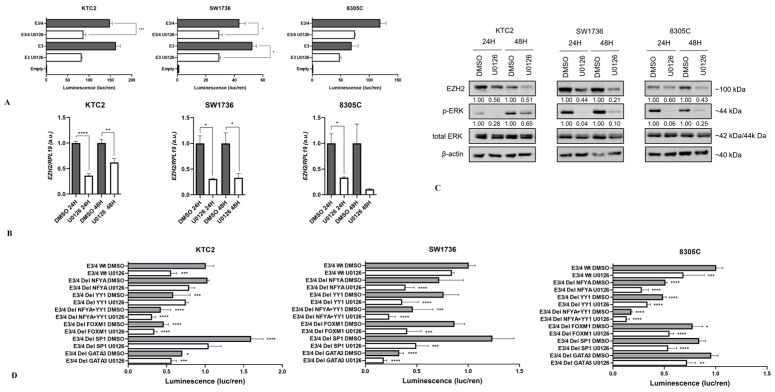
*EZH2* activation is controlled by MAPK signaling in ATC. (**A**): The inhibition of MAPK signaling with 10 µM of U0126 reduced the *EZH2* promoter activity measured by E3/4 luciferase reporter plasmid and (**B**): reduced *EZH2* transcription in qPCR analysis after 24 h and 48 h of treatment. Luciferase activity was normalized using Renilla luminescence. The gene expression was normalized using *RPL19*. a.u., arbitrary unit. Data represented as mean ± SD (*n* = 3). (**C**): MAPK signaling blockage also reduced EZH2 protein levels in all ATC cell lines. Treatment effectiveness was validated by the reduction in ERK phosphorylation after treatment for 24 and 48 h. The protein expression was normalized using β-actin. The drug-treated samples were normalized to DMSO levels and showed as fold-change. (**D**): Analysis of the effect of MAPK inhibition on E3/4 plasmid containing TF deletion showed an additive effect of *EZH2* promoter activity reduction. Luciferase activity was normalized using Renilla luminescence. Data represented as mean ± SD (*n* = 3). * *p* < 0.05; ** *p* < 0.01; *** *p* < 0.001; **** *p* < 0.0001 vs. DMSO control.

The in silico prediction for the E3/4 region showed binding sites for TFs related to MAPK signaling, like NFYA, SP1, FOXM1 and GATA3 (Table S3). We then analyzed whether *EZH2* induction is linked to MAPK activation in ATC cells. For this, we treated ATC cell lines with U0126, an MEK1/2 inhibitor, which results in reduction in ERK1/2 phosphorylation, an effector of MAPK pathway. After MEK1/2 inhibition, we observed a significant reduction in E3/4 promoter luminescence (Figure 3A), as well as a reduction in *EZH2* mRNA levels in ATC cell lines (Figure 3B), indicating that *EZH2* transcriptional activation is dependent on MAPK. We also observed a significant reduction in ERK phosphorylation and EZH2 protein levels in ATC cells, which indicates that EZH2 protein expression is dependent on MAPK activation (Figure 3C).

We then analyzed if the MEK1/2 inhibition could synergize with TF site deletion in the luciferase assay. We observed significant reduction in luminescence in the cells treated with U0126, when compared to the effect of the deletions taken alone (Figure 3D).

**Figure 4:**
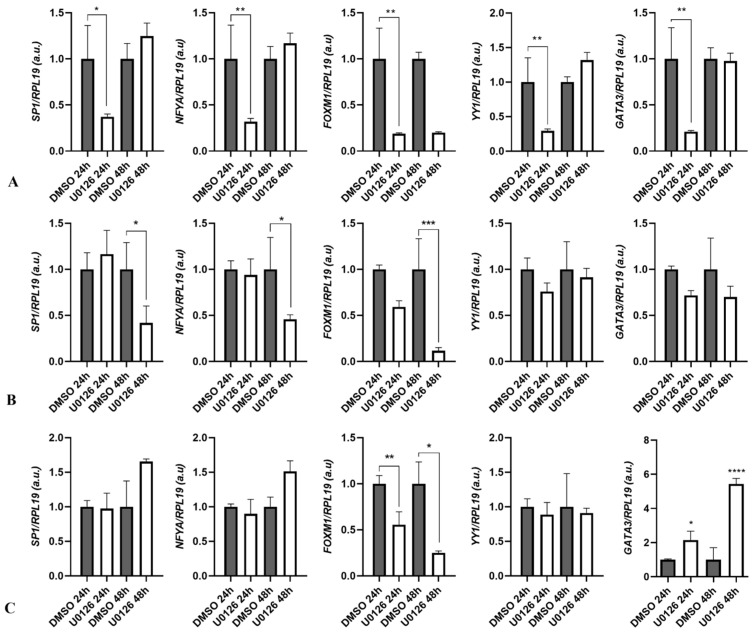
TFs’ gene expression after MAPK inhibition with U0126 in ATC cells. (**A**): *NFYA*, *YY1*, *SP1*, *FOXM1* and *GATA3* expression in KTC2 cells. (**B**): TF expression in SW1736 cells. (**C**): TF expression in 8305C cells. Gene expression was normalized using the comparison between the U0126-treated cells and control cells (U0126/control DMSO). *RPL19* levels were used as endogenous control. Data represented as mean ± SD (*n* = 3). a.u., arbitrary unit. * *p* < 0.05; ** *p* < 0.01; *** *p* < 0.001; **** *p* < 0.0001 vs. DMSO control.

Next, to assess TFs that could be downstream effectors of the MAPK pathway, we analyzed the gene expression of *NFYA*, *YY1*, *SP1*, *FOXM1* and *GATA3*—after U0126 treatment in ATC cells. As a result, we observed a strong reduction in *FOXM1* expression in response to MEK1/2 inhibition in all ATC cell lines analyzed after 24 and 48 h of treatment, while the other TFs showed variable expression modulation at a single time point (Figure 4A–C).

**Figure 5:**
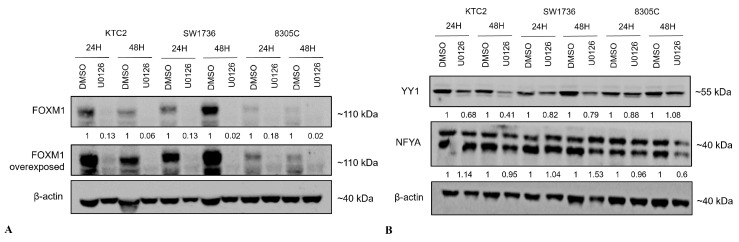
Effect of MAPK inhibition on TF expression. (**A**): The blockage of MAPK signaling with U0126 treatment reduced the protein levels of FOXM1 after 24 h and 48 h compared to control. (**B**): YY1 protein levels were also reduced after MAPK signaling inhibition. The protein expression was normalized using β-actin. The drug-treated samples were normalized to DMSO levels and showed as fold-change.

Additionally, we analyzed the protein expression of NFYA, YY1 and FOXM1 after MAPK signaling inhibition (for comparison, basal expression levels in thyroid cancer cell lines are shown in Figure S3). We observed a strong reduction in FOXM1 upon U0126 treatment in both times analyzed, while YY1 showed a less expressive reduction in cells treated with U0126 (Figure 5A,B). On the other hand, NFYA protein levels remained relatively unaffected in KTC2, while we observed opposed effects in SW1736 and 8305C cells after 48 h (Figure 5B).

### 2.4. Effects of NFYA, YY1, and FOXM1 Overexpression on EZH2 Expression

**Figure 6:**
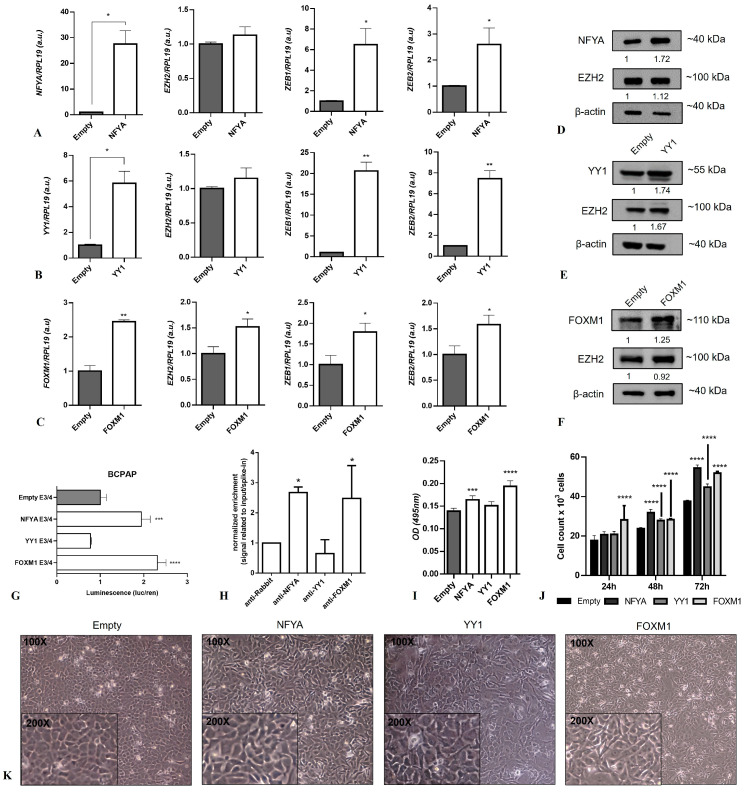
Effect of TF overexpression in BCPAP cell line. Overexpression of NFYA (**A**), YY1 (**B**) and FOXM1 (**C**) was validated by qPCR using primers for the TFs, *EZH2*, and EMT-TFs *ZEB1* and *ZEB2*. The gene expression was normalized using *RPL19*. Data represented as mean ± SD (*n* = 3). Validation of NFYA (**D**), YY1 (**E**) and FOXM1 (**F**) overexpression by WB. Expression was normalized using β-actin and overexpression samples were normalized to empty control levels and showed as fold-change. (**G**): *EZH2* luciferase promoter activation (E3/4 fragment) in response to TF overexpression. Luciferase activity was normalized using Renilla luminescence. Data represented as mean ± SD (*n* = 3). (**H**): CUT & RUN analysis using SW1736 cells show enrichment for NFYA and FOXM1 TFs in E3/4 region of EZH2 promoter. Enrichment for each TF is calculated as related to input/spike-in levels following kit protocol. Data represented as mean ± SD (*n* = 3). a.u., arbitrary unit * *p* < 0.05 vs. control (anti-Rabbit). Functional effects of TF overexpression in BCPAP cell viability (**I**) measured by MTT assay. Data represented as mean ± SD (*n* = 8). Effect on cell counting (**J**) after 24 h, 48 h and 72 h. Data represented as mean ± SD (*n* = 3). a.u., arbitrary unit. * *p* < 0.05; ** *p* < 0.01; *** *p* < 0.0001; **** *p* < 0.0001 vs. Empty control. Effect on cell morphology (**K**) under phase contrast microscopy shows a change in cell morphology towards a mesenchymal-like phenotype after TFs overexpression.

Our data shows that ATC cells express higher levels of *NFYA*, *YY1* and *FOXM1* compared to PTC cells (Figure 2A). Thus, we overexpressed these TFs in the PTC cell line BCPAP to investigate their influence on EZH2 activation in ATC. First, we validated the overexpression of NFYA, YY1 and FOXM1 in BCPAP cells by qPCR, and observed that only FOXM1 overexpression induced *EZH2* mRNA. Moreover, overexpression of these TFs increased the expression of two main EMT-TFs, *ZEB1* and *ZEB2* (Figure 6A–C). Next, we accessed the protein levels of TFs and EZH2 and observed that only YY1 overexpression induced EZH2 in BCPAP cells (Figure 6D–F). *EZH2* promoter activation (E3/4 plasmid) was observed only in NFYA and FOXM1 overexpressing cells compared to the BCPAP control (Figure 6G). Additionally, we quantified the levels of miR-25-3p, an miRNA that target *EZH2* mRNA and observed an increase in miR-25-3p expression in BCPAP with NFYA and FOXM1 overexpression, while no changes were detected in YY1 overexpression (Figure S4).

To investigate NFYA, YY1 and FOXM1’s direct interaction with the *EZH2* promoter region, we performed CUT & RUN assay using specific antibodies against each TF. The results showed enrichment for NFYA and FOXM1 TFs in the E3/4 region of *EZH2* promoter in SW1736 ATC cells that express high levels of these TFs (Figure 6H).

Then, we performed functional assays to investigate the effects of NFYA, YY1 and FOXM1 overexpression in PTC biology. The MTT assay showed significant increase in cell viability with NFYA and FOXM1 overexpression (Figure 6I), while the three TF overexpression increased the cell number compared to control (Figure 6J), and altered cell morphology to a mesenchymal-like phenotype (Figure 6K).

### 2.5. FOXM1 Knock-Down Reduces EZH2 Activation in ATC

**Figure 7:**
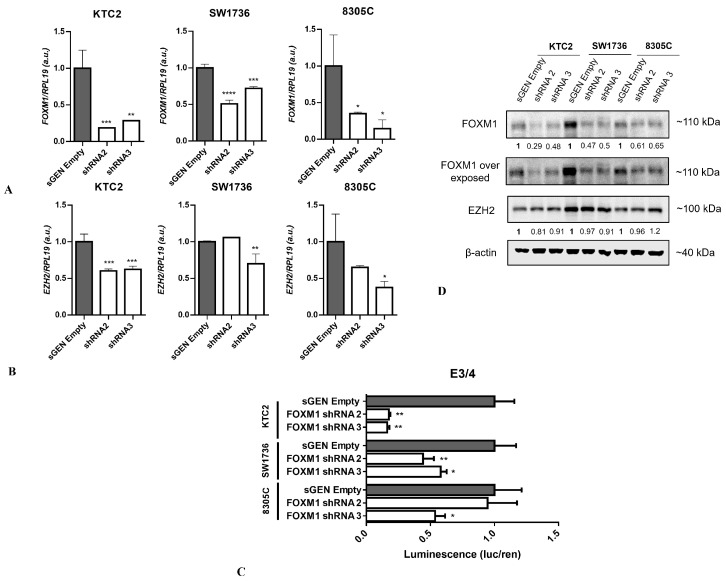
Effect of FOXM1 modulation in ATC cells. (**A**): Knock-down of *FOXM1* was performed using two shRNA plasmids (sh2 and sh3) and resulted in significant decrease in *FOXM1*. (**B**): *EZH2* expression was reduced after *FOXM1* knock-down in ATC cell lines. The gene expression was normalized using *RPL19*. (**C**): The activation of *EZH2* minimal promoter E3/4 was reduced after *FOXM1* interference in ATC cells. Luciferase activity was normalized using Renilla activity. Data represented as mean ± SD (*n* = 3). a.u., arbitrary unit; * *p* < 0.05; ** *p* < 0.01; *** *p* < 0.001; **** *p* < 0.0001 vs. sGEN Empty control. (**D**): *FOXM1* knock-down reduced FOXM1 protein levels in all cell lines, while EZH2 protein expression was mostly unaltered.

Next, we focused on FOXM1 as it is overexpressed in ATC and shows greater response to MAPK signaling inhibition compared to the other TFs (Figure 4 and Figure 5). In order to knock-down *FOXM1* mRNA, we transfected ATC cells with a plasmid that overexpresses an shRNA against *FOXM1* using miR-E methodology. As result, we observed a significant reduction in mRNA levels of *FOXM1* expression in ATC cells, particularly with shRNA 2 and shRNA 3 (Figure 7A). Subsequently, we assessed the effect of *FOXM1* knock-down in EZH2 expression and observed a reduction in *EZH2* mRNA levels by qPCR (Figure 7B), and reduction in E3/4 promoter activation in ATC cells (Figure 7C). FOXM1 protein expression was also reduced in shRNA 2 and shRNA 3 cell lines, but only a slight effect in EZH2 protein levels was observed mostly in KTC2 cells (Figure 7D).

## 3. Discussion

EZH2 is the main component of the PRC2 chromatin silencing complex and is overexpressed in ATC [22]. We have shown previously that modulation of EZH2 function in ATC cells using CRISPR/Cas9 and the inhibitor EPZ6438 results in antitumor effects and induces cell redifferentiation [15]. These results indicate that EZH2 is involved in the acquisition of aggressive behavior and thyroid cancer progression.

In our study, we investigated the activation of the human *EZH2* promoter and identified the minimal promoter region as a small fragment with ~107 bp, termed here as the E3/4 region, within the −102 +5 region from *EZH2* TSS. TF prediction analysis showed that several TFs can bind to this region, specifically NFYA, YY1, SP1, FOXM1 and GATA3, whose deletion resulted in reduction in EZH2 promoter activity. Moreover, these TFs are overexpressed in ATC cell lines, correlating with *EZH2* transcriptional activation in ATC.

Indeed, the TFs investigated in our study are implicated in cancer progression, and our study further supports their role in ATC biology. For instance, NFYA has been shown to contribute to tumor growth and cell proliferation in breast cancer cells [24], and it has been associated with the activation of *EZH2* promoter in ovarian cancer. On the other hand, FOXM1 is known for its role in controlling cell proliferation, invasion and angiogenesis [25] and has been implicated in PTC and ATC progression [26,27]. GATA3 is a new TF detected in ATC, which is expressed in breast cancers [28] and serous ovarian carcinomas [29]. This TF is normally associated with differentiation of luminal breast cells [30], while it was also described as an angiogenesis inductor and recruiter of tumor-associated macrophages. YY1 is highly expressed in thyroid cancer cells compared to non-tumoral thyroid cells [31], implicating its contribution to thyroid carcinogenesis and progression. Intriguingly, YY1 can recruit EZH2 to gene promoters in a noncanonical binding and induce EZH2-mediated gene silencing through methylation deposition in breast cancer [32].

Additionally, E2F1, a positive regulator of the cell cycle, can activate EZH2 in the bladder cancer context [33], increasing cell proliferation. Indeed, our data show that E2F1 expression is higher in ATC cells, but deletion of E2F1 site did not change luciferase activation, indicating that E2F1 may not directly activate *EZH2* in ATC. Another TF identified in the *EZH2* minimal promoter region is SP1, essential for inducing the transcription of cell maintenance genes and a downstream effector of MAPK signaling [34], which is overactivated in ATC cells.

Indeed, it was described that EZH2 responds to MAPK inhibition in breast, lung, colon and pancreatic cancer [35,36]. We found that blocking MAPK activation with U0126 inhibitor reduced the transcriptional levels of *EZH2*, its promoter activation, as well as its protein levels, indicating that *EZH2* activation depends on an active MAPK induction in ATC. Moreover, we observed that FOXM1 and YY1 expression are dependent on MAPK activation, suggesting these TFs may act as MAPK effectors in ATC. Indeed, studies report that NFYA and FOXM1 are activated by MAPK signaling via ERK1/2 phosphorylation, acting as MAPK effectors in the cell nucleus [37,38]. Nevertheless, when combining TF site deletion with U0126 treatment, we observed an additive effect on *EZH2* promoter activity repression, which implicates that MAPK signaling is the main upstream controller of the TFs that regulate *EZH2* promoter activity.

Finally, we showed that overexpression of TFs in thyroid cancer cell lines induces *ZEB1* and *ZEB2* expression, suggesting the activation of EMT in BCPAP cells, while affecting EZH2 levels. The overexpression of YY1 increased EZH2 protein in BCPAP, without inducing luciferase reporter activation, which indicates that YY1 may modulate EZH2 protein levels through a non-promoter interaction, modulating mRNA stability or post-translational mechanism that enhances protein translation/stability. On the other hand, both NFYA and FOXM1 overexpression activated the E3/4 reporter plasmid, indicating a direct binding to the *EZH2* promoter, although there was a significant increase in *EZH2* mRNA levels only in FOXM1 overexpression. In this context, we used CUT&RUN assay to investigate NFYA, YY1 and FOXM1 binding to the *EZH2* promoter in ATC cells, which revealed an enrichment in the E3/4 region only for NFYA and FOXM1. These results indicate that NFYA and FOXM1 can bind to E3/4 region and activate *EZH2* transcription.

Moreover, the overexpression of TFs point to an miRNA-mediated regulation of EZH2 levels in BCPAP cells. For example, FOXM1 overexpression induces *EZH2* promoter activation and mRNA expression without increasing protein levels, indicating the participation of miRNAs in modulating EZH2 translation, or NFYA overexpression induces *EZH2* promoter activity without increasing its mRNA or protein levels, suggesting the participation of miRNAs in regulating *EZH2* mRNA stability and translation. Indeed, miRNAs can potentially regulate EZH2 protein levels by interacting with its short 3′UTR (<300 nt in length). For example, it was shown that downregulation of miR-25 and miR-30d derepress *EZH2* mRNA translation and induce its overexpression in ATC [16]. Indeed, we showed that miR-25 expression is increased in NFYA and FOXM1 overexpressing cells, indicating that this miRNA can repress EZH2 protein translation in this case, an effect not observed in YY1 overexpression.

Lastly, *FOXM1* knock-down in ATC cell lines reduced *EZH2* mRNA and E3/4 luciferase promoter activation, indicating that FOXM1 regulates *EZH2* levels transcriptionally. However, the absence of EZH2 protein levels modulation in response to *FOXM1* knockdown points to enhanced EZH2 protein stability and the participation of other molecular mechanisms that maintain EZH2 levels in ATC.

We reckon that our study has some methodological limitations regarding how luciferase assays artificially mimic the real transcriptional process, mostly in cases that *EZH2* mRNA levels do not reflect E3/4 promoter activation. Nevertheless, our study brings important insights into the regulatory network of TFs and MAPK for *EZH2* activation in ATC. The individual modulation of these TFs may somehow influence *EZH2* promoter, mRNA or protein in PTC cells, but we extrapolate that the overall effect observed in ATC may result from the combinatorial overexpression of all TFs allied to the upstream activation MAPK signaling, downstream miRNA modulation and additional mutations such as in *TP53*. Altogether, the resulting effect is the sustained activation of *EZH2* in ATC leading to the epigenetic deregulation that cannot be counteracted, for example, by a single knock-down of *FOXM1*, as *EZH2* activation is multifactorial.

**Figure 8:**
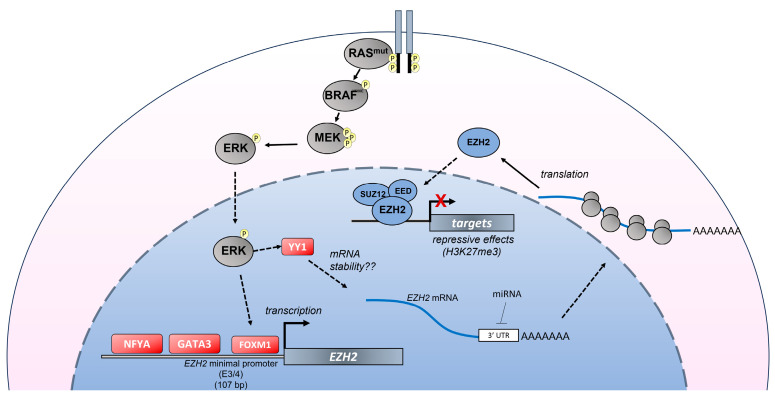
Graphical abstract. Regulatory network that controls *EZH2* minimal promoter region (E3/4) is composed of MAPK-dependent and independent transcription factors that mainly activate *EZH2* transcription but may also influence *EZH2* mRNA stability in anaplastic thyroid cancer. EZH2 overexpression leads to enhanced PRC2 complex activity and target gene repression by epigenetic deposition of H3K27Me3 marker.

Hence, in this study, we identified the minimal promoter region of the human *EZH2* gene (−102 +5 from EZH2 TSS), responsible for its transcriptional activation in ATC cells. We also showed that this region is responsive to MAPK signaling and to transcription factors that crosstalk with MAPK signaling such as NFYA, YY1, SP1, FOXM1 and GATA3, comprising a new regulatory network for *EZH2* sustained activation in ATC (Figure 8). Thus, further investigation about the effects of targeting these network nodes individually or in combination could yield valuable insights for the understanding of ATC biology.

## 4. Materials and Methods

### 4.1. Cell Lines, Culture and Treatments

Human-derived thyroid cancer cell lines were used in this study. We used three ATC cell lines: KTC2, SW1736 and 8305C, and two PTC cell lines, BCPAP and TPC-1. All cell lines were kept at 37 °C in an incubator with CO2 at 5%, and culture medium conditions described in Table S1. The cell lines TPC-1, BCPAP and SW1736 were kindly provided by Dr. James Fagin (Memorial Sloan Kettering Cancer Center, USA). KTC2 cell line was kindly provided by Dr. Norisato Mitsutake (University of Nagasaki, Japan). All the cell lines used in this study were authenticated by Short Tandem Repeat (STR) analysis following the ANSI Standard ASN-0002 (2012). Sixteen STR loci plus the gender-determining locus Amelogenin were amplified using the commercially available PowerPlex^®^ Fusion 6C Kit (Promega, Madison, WI, USA). Samples were processed on an ABI 3130 Genetic Analyzer (Thermo Fisher Scientific, Waltham, MA, USA), and data were analyzed with GeneMarker^®^ hid software v 2.8.2 (SoftGenetics, State College, PA, USA) and compared with the DSMZ and Cellosaurus databases. Also, all experiments were performed using mycoplasma-free cells.

For MAPK signaling inhibition, cells were treated with 10 µM of the MEK1/2 inhibitor U0126 (Promega, Madison, WI, USA, cat. V1121) diluted in DMSO and added to the culture medium for 24 h and 48 h, when total RNA and protein were extracted. In the luciferase reporter assays, cells were pre-treated with U0126 for 1 h in Opti-MEM medium prior to plasmid transfection with Lipofectamine 2000TM (Invitrogen, Thermo Fisher Scientific, Waltham, MA, USA, cat. 11668027), and U0126 was added again when the medium was changed for complete medium 4 h after the transfection. As control, cells were treated with the same amount of DMSO (vehicle).

### 4.2. Analysis of EZH2 Promoter Activity by Luciferase Assay

First, we used Genome Browser (https://genome.ucsc.edu/) (accessed on 10 October 2021) to identify the most active region of EZH2 gene promoter around exon1, which contains the highest H3K27ac levels and indicates where the chromatin is open and active for transcription. We designed primers with Primer Blast (https://www.ncbi.nlm.nih.gov/tools/primer-blast/) (accessed on 10 October 2021) to amplify fragments of *EZH2* promoter region initially into 5 segments: E1 (−1540 −1069), E2 (−1143 −503), E3 (−504 +5), E4 (−102 +334) and E5 (+577 +1109) (Table S2). We also designed primers that fragmented E3 and E4 into 3 smaller fragments, E3A (−504 −304), E3B (−303 −110) and E3/4 (−102 +5) (Table S2), which consists of the overlapping region between E3 and E4. Primers contain sites for XhoI (Fw) and BglII (Rv) enzymes for cloning in pGL4-20 plasmid.

We cloned *EZH2* promoter fragments using the genomic DNA extracted from non-tumoral human thyroid Nthy-ori3-1 as template (100 ng) in a conventional PCR reaction containing 500 nM of primers, 0.2 mM dNTPs, 1.5 mM MgCl2 and Taq Polymerase for 35 cycles at 55 °C annealing. For more difficult amplifications the annealing temperatures were changed, as we added DMSO (final concentration at 5%) or betaine (final concentration of 1M) in the PCR reaction. The PCR reaction was purified with Qiack Gel Extraction Kit (Qiagen, Hilden, Germany, cat. 28704) and digested with XhoI and BglII overnight at 37 °C. Then, the digested fragments were ligated into pGL4-20 minP luciferase reporter plasmid, which was double digested with XhoI + BglII, and transformed in DH5α bacteria. The plasmids were then validated by Sanger sequencing and used in cell transfection.

For the analysis of *EZH2* promoter, we constructed 8 different plasmids, one for each *EZH2* promoter fragment (E1 to E5, E3A, E3B and E3/4), plus the control plasmid without any promoter fragment inserted (pGL4-20 minP-empty), that were transfected into TPC-1, BCPAP, KTC2, SW1736 and 8305C lines. pGL4-20 plasmids were co-transfected with the pRL plasmid which transcribes constitutively the Renilla luciferase used to normalize transfection efficiency in the Dual Luciferase assay.

For that, 5 × 10^4^ cells were plated in 24-well plates in triplicates, using complete medium. After 24 h, cells were placed in Opti-MEM medium and transfected with 300 ng of pGL4-20 + 30 ng pRL (1:10 ratio) using Lipofectamine 2000TM (Invitrogen) in the proportion of 1:2 (DNA:lipofectamine2000). After 4 h of transfection, the medium was changed to a complete medium. Twenty-four hours after transfection, the cell lysate was collected using 1× Passive Lysis Buffer (Promega, Madison, WI, USA, cat. E1500) and luminescence was measured using Dual Luciferase^®^ Reporter Assay kit (Promega) in the luminometer GloMax (Promega).

### 4.3. Site-Directed Deletion of Transcription Factors Binding Sites

We used the LASAGNA online algorithm (https://biogrid-lasagna.engr.uconn.edu/lasagna_search/index.php) (accessed on 21 October 2021) for in silico prediction of potential transcription factors (TFs) that could bind to the *EZH2* minimal promoter region. All 5 databases (JASPAR CORE, TRANSFAC, UniPROBE, ORegAnno and PAZAR) available were used in the search, and the TFs with higher scores were chosen for further analysis (Table S3).

Site-specific deletions of TF binding sites (Table S4) were performed using Q5 Site-Directed Mutagenesis Kit (New England BioLabs, Ipswich, MA, USA, cat. E0554S) and primers (Table S4) designed in NEBase Changer website (https://nebasechanger.neb.com/) (accessed on 20 July 2022). For the site alteration reactions, we used E3/4 pGL4-20 minP plasmid as the template and performed a PCR reaction using the Q5 Hot Start High-Fidelity mastermix, with 25 cycles and a specific setup of temperature depending on the annealing temperature of primers. The PCR reaction was followed by a Kinase, Ligase & DpnI (KLD) treatment for the circularization of the PCR product and degradation of template plasmid. Next, the KLD product was transformed in highly efficient competent bacteria, and clones were purified by QIAprep Spin Miniprep Kit (Qiagen). We constructed one plasmid for each TF site deletion for NFYA, YY1, SP1, FOXM1, E2F1 and GATA3, plus plasmids with TF binding site double deletions for NFYA + YY1. The alterations were confirmed by Sanger sequencing and the plasmids were used for cell transfection as described previously.

### 4.4. FOXM1 Knockdown by shRNA

We used the miR-E methodology [39] to stably produce shRNA to knockdown *FOXM1* in ATC cells, which showed higher protein expression of FOXM1 (Figure S3). Short hairpin RNAs were designed using the splash RNA online program (http://splashrna.mskcc.org/) (accessed on 18 March 2023) and the 3 sequence guides that were closer to the start of the coding sequence of the gene were chosen for cloning (Table S5). We first diluted the oligos (97-mer) to 1000 ng/µL and after that, serially diluted the oligos to a final concentration of 0.05 ng/µL to perform a PCR reaction using miR-E primers (miR-XhoI-fw and miRE-EcoOligo-Rv) (Table S2) and SuperFi polymerase (Invitrogen, cat. 12351010) to amplify the oligos. After validation of the amplification (141 bp fragment) using a 2% agarose gel, the PCR reaction was purified with Qiack Gel Extraction Kit, digested with XhoI and EcoRI for 4 h at 37 °C and ligated into sGEN plasmid previously digested with XhoI and EcoRI. SGEN was a gift from Johannes Zuber (Addgene plasmid # 111171). The ligations were transformed into DH5α bacteria and plasmids were extracted using the QIAprep Spin Miniprep Kit (Qiagen). After validation by Sanger sequencing, the plasmids were transfected into ATC cells that were selected with G418 treatment for 12 days (400 µg/mL for KTC2 and 8305C and 600 µg/mL for SW1736) to generate stable populations that were used for validation of FOXM1 knockdown by qPCR and Western blotting.

### 4.5. Gene Expression Analysis

Total RNA was phenol–chloroform-extracted from thyroid cancer cell lines using TRIzol (Invitrogen) according to the manufacturer’s instructions. Protein coding gene expression was investigated using cDNA generated from the reverse transcription of 3 µg of total RNA using oligo-dT primer, random primers and MMLV reverse transcriptase (Invitrogen). Quantitative PCR was performed in a Viia7^®^ Sequence Detection System (Applied Biosystems, Thermo Fisher Scientific, Waltham, MA, USA, cat. 4453545) using SYBR Green master mix (Applied Biosystems cat. 4309155), cDNA and specific primers for *FOXM1*, *NF1*, *YY1*, *NFYA*, *KLF4*, *E2F1*, *SP1*, *GATA3*, *ZEB1*, *ZEB2*, *RPL19* and *EZH2* (Table S6). Gene expression was normalized by RPL19 levels and calculated using the QGENE program.

To evaluate the expression of microRNAs (miRNAs), 10 ng of total RNA were subjected to reverse transcription using the TaqMan^®^ Reverse Transcription Kit (Applied Biosystems, Thermo Fisher Scientific, Waltham, MA, USA), in the presence of stem-loop primers for microRNAs. Next, qPCR was performed using TaqMan MicroRNA Assays kits for miR-25-3p (assay 403), miR-30d-5p (assay 420), and U6 (assay 1973), on the ViiA7^®^ Sequence Detection System (Applied Biosystems, Thermo Fisher Scientific, Waltham, MA, USA). miRNA expression was normalized by comparison with U6 levels and calculated using the Qgene program.

### 4.6. Western Blotting Analysis

Total protein was extracted from thyroid cancer cells using a RIPA buffer containing 10% protease inhibitor cocktail and 1% phosphatase inhibitor cocktail (Sigma, St. Louis, MO, USA). Protein concentration was determined using Bradford (Bio-Rad Laboratories, Hercules, CA, USA, cat. 5000205), and 30 µg of each sample was fractionated by 10–12% SDS-PAGE and blotted onto a Hybond-ECL nitrocellulose membrane (Amersham Biosciences, Little Chalfont, UK, cat. RPN3032D). Nonspecific binding sites were blocked with 5% nonfat dry milk in PBS—0.1% Tween-20 (Merck KGaA, Darmstadt, Germany, P9416). The following primary antibodies were used as specified in Table S7: anti-EZH2 (Cell Signaling, Danvers, MA, USA, cat. #4905), anti-pERK (Santa Cruz Biotechnology, Dallas, TX, USA, #SC 7383), anti-ERK1/2 (Santa Cruz Biotechnology, cat. #SC 94), anti-NFYA (Thermo, cat. #MA5-36198), anti-YY1 (Thermo cat. 712089), anti-FOXM1 (Cell Signaling, cat. #5436) and anti-β-Actin (Santa Cruz Biotechnology, cat. #SC 47778), as an endogenous control. Immunoexpression was detected with horseradish peroxidase-conjugated secondary antibodies (GE Healthcare, Little Chalfont, UK, cat. RPN1231) and developed with luminol and p-coumaric acid (Sigma Aldrich, cat. 722812) reagents in the presence of H2O2. Chemiluminescence emission was visualized in an ImageQuant LAS4000 imaging system (GE Healthcare) and band intensities were quantified with ImageJ software v.2.16.0.

### 4.7. Overexpression of Transcription Factors (TFs)

We used MSCV-Puro plasmid to clone the coding sequence of isoform 1 of *NFYA* (1.045 bp), *YY1* (1.244 bp) and *FOXM1* (2.232 bp) amplified by PCR (Table S2) using the cDNA from SW1736 cell line as template. MSCV Puro was a gift from Tyler Jacks (Addgene plasmid # 68469). For *YY1* amplification we used betaine (final concentration of 1M) in the PCR reaction at 60 °C, while for *NFYA,* amplification the PCR reaction was performed at 59 °C, and for *FOXM1*, at 55 °C. The PCR inserts were digested with XhoI and EcoRI and ligated into the same sites of MSCV-puro plasmid. The plasmids were transformed into DH5α bacteria and extracted using the QIAprep Spin Miniprep Kit (Qiagen). After confirmation by Sanger sequencing, the plasmids were transfected in PTC cell line BCPAP and stable cell lines were generated using puromycin (1ug/mL) selection for one week. BCPAP cell line was chosen based on YY1 and FOXM1 protein expression, which was lower than in ATC cells (Figure S3). Overexpression of NFYA, YY1 and FOXM1 was validated by qPCR and Western blotting.

### 4.8. CUT & RUN Assay

The Cleavage under Targets and Release Using Nuclease (CUT&RUN) assay was performed as described previously [23]. For this work, we used 1 × 10^5^ SW1736 cells per immunoprecipitation (IP), including 3 IP’s for each TF (NFYA, YY1 and FOXM1), 1 IP for negative antibody control (Rabbit) and 1 IP for the nonprecipitated control (input). The primary antibodies were used as follows: 8 μL of anti-NFYA (Invitrogen—PA5-84030), anti-YY1 (Invitrogen—712089), anti-FOXM1 (Cell signaling—5436), and 5 μL of anti-IgG Rabbit (663625—negative control). qPCR was performed to detect enrichment in the E3/4 region of *EZH2* promoter using specific primers (Table S8). Enrichment was calculated using the following formula: Percent Input = 100% × 2(C[T] 100% Input Sample—C[T] IP Sample), as described in the kit. Enrichment values were then normalized using spike-in control to calculate the normalization factor. The graph values were then calculated through dividing enrichment levels per normalization factor values.

### 4.9. Cell Counting

For cell counting, 2.5 × 10^4^ cells per well were plated into 12-well plates in triplicates and grown for 24, 48 and 72 h. After these periods, cells were washed with PBS (ph7.4), detached with 0,25% trypsin (Thermo) diluted in PBS (ph7.4) and fixated with 3.7% formaldehyde diluted in PBS (ph7.4). Cells were then counted using Guava Easycyte Mini cytometer (Millipore, Burlington, MA, USA).

### 4.10. MTT Assay

For MTT assay, 5 × 10^3^ cells per well were plated into a 96-well plate in 8 replicates and grown for 24 h. Next, 10 µL of 2.5 mg/mL MTT (3-(4,5-dimethylthiazol-2-yl)-2,5-diphenyltetrazolium bromide) (Invitrogen, cat. M6494) diluted in water was added per well, and after that the cells were kept at 37 °C for 3 h. After incubation, the media was removed and 100 µL of 0.04 M isopropanol/HCl was added per well. Next, absorbance was measured using a spectrophotometer at 595 nm.

### 4.11. Gene Correlation and Survival Prognosis Analysis in GEPIA2 Database

The GEPIA2 (Gene Expression Profiling Interactive Analysis 2) database (http://gepia2.cancer-pku.cn/) (accessed on 8 May 2024) [40] was used to search for correlation between *EZH2* and TF expression, and their survival prognosis significance in a dataset of 521 patients with thyroid cancer (THCA) and 337 control patients from The Cancer Genome Atlas (TCGA).

To assess gene correlation, *EZH2* was paired with each TF, and the Pearson correlation coefficient (R value) was obtained. Interpretation was as follows: less than 0 (negative correlation), 0 to 0.25 (weak positive correlation), 0.25 to 0.50 (moderate positive correlation), 0.50 to 0.75 (strong positive correlation), and 0.75 to 1 (very strong positive correlation) at *p*-value <0.05.

For survival analysis, we used the “Survival Analysis-Survival Map” to obtain disease-free survival (DFS) and overall survival (OS) data of THCA of 521 patients with thyroid cancer and 337 control patients from The Cancer Genome Atlas (TCGA). To obtain the expression threshold significance cohorts, we used the median 50% cutoff-high and 50% cutoff-low at a *p*-value of 0.05. Kaplan–Meier curves were generated using the “Survival Analysis” to visualize survival outcomes for cohorts exhibiting significant expression thresholds including Hazard Ratios and 95% confidence interval sets.

### 4.12. Statistical Analysis

The results were presented as the mean ± standard deviation (SD) and were submitted to ANOVA analysis of variance followed by a *t*-test or Tukey’s test. Differences were considered significant at *p* < 0.05.

## Data Availability

The original contributions presented in this study are included in the article/Supplementary Materials. Further inquiries can be directed to the corresponding author.

## Supplementary Materials

The following supporting information can be downloaded at: https://www.mdpi.com/article/10.3390/ijms27146402/s1.

## Abbreviations

The following abbreviations are used in this manuscript:

TFs	Transcription Factors
ATC	Anaplastic Thyroid Carcinoma
PTC	Papillary Thyroid Carcinoma
NIS	Sodium Iodide Symporter
PRC2	Polycomb Repressive Complex 2
