# Supplementary material for: Activation of EZH2 Promoter Is Mediated by MAPK Signaling Regulated Transcription Factors in Anaplastic Thyroid Cancer
Source: Int J Mol Sci. 2026 Jul 18;27(14):6402. doi: 10.3390/ijms27146402 (PMC13410663; doi:10.3390/ijms27146402)
Supplement: Supplementary file 1 [file ijms-27-06402-s001.zip › ijms-4385210-supplementary.pdf]

SUPPLEMENTARY DATA

Supplementary Figures

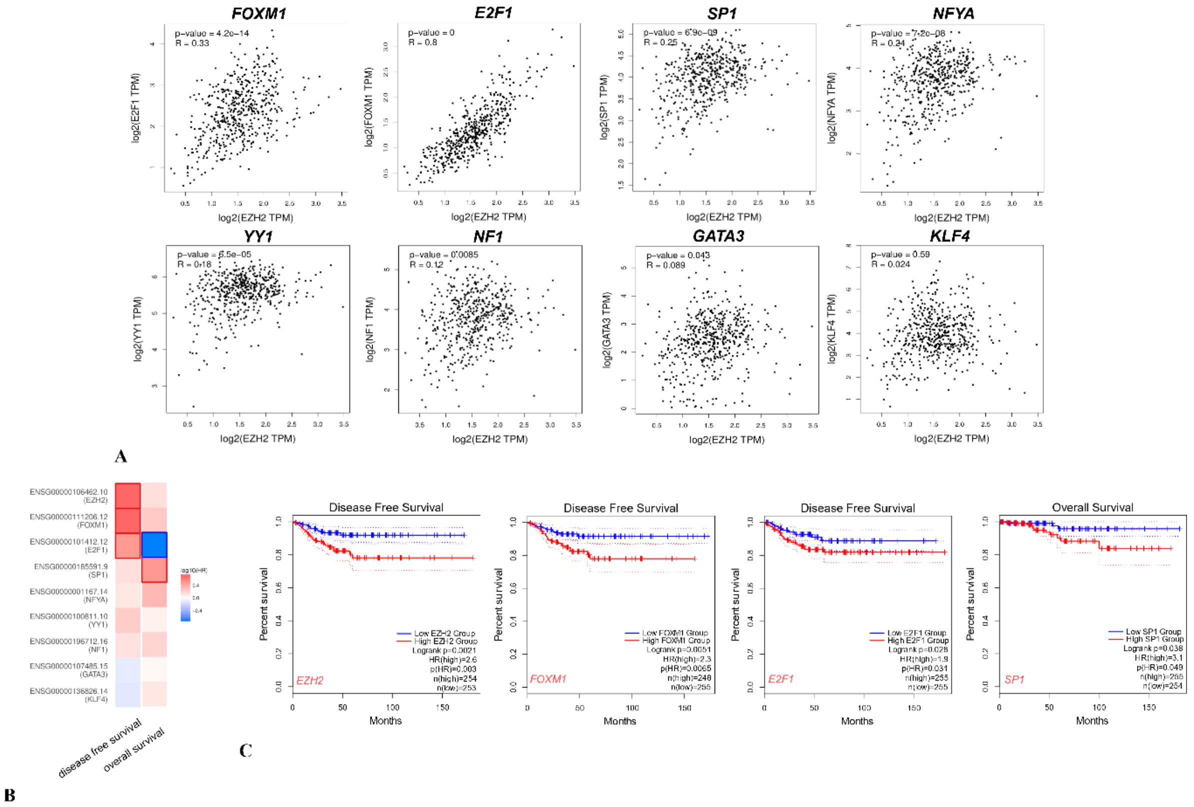

Supplementary Figure S1. Data from the GEPIA 2 (Gene Expression Profiling Interactive Analysis) platform shows that TFs overexpression correlates with *EZH2* gene expression and with decreased patient survival in thyroid cancer. (A): Positive correlation between TFs and *EZH2* expression levels in thyroid cancer. (B): Highlighted red spots indicate significant relation between higher expression of *EZH2*, *E2F1* and *FOXM1* and lower disease- free survival rates of patients with thyroid cancer. The data also show significance for the relation between higher *SP1* expression and overall survival rate of patients with thyroid cancer. (C): The survival analysis graphs indicate decrease in disease free survival rates for patients that present higher expression levels of *EZH2*, *E2F1* and *FOXM1* and decrease in overall survival rates for patients that present higher expression levels of *SP1*.

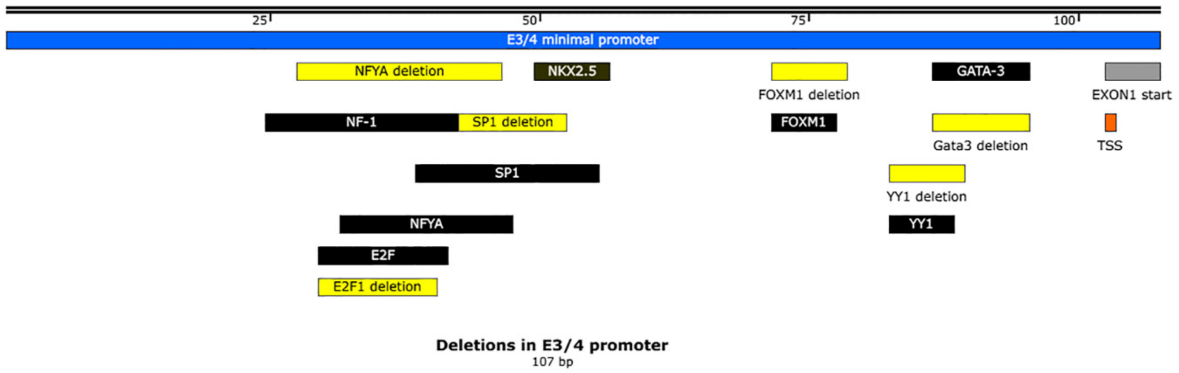

Supplementary Figure S2. Schematic view of E3/4 minimal promoter showing TFs binding sites and the respective deletions in the luciferase plasmids.

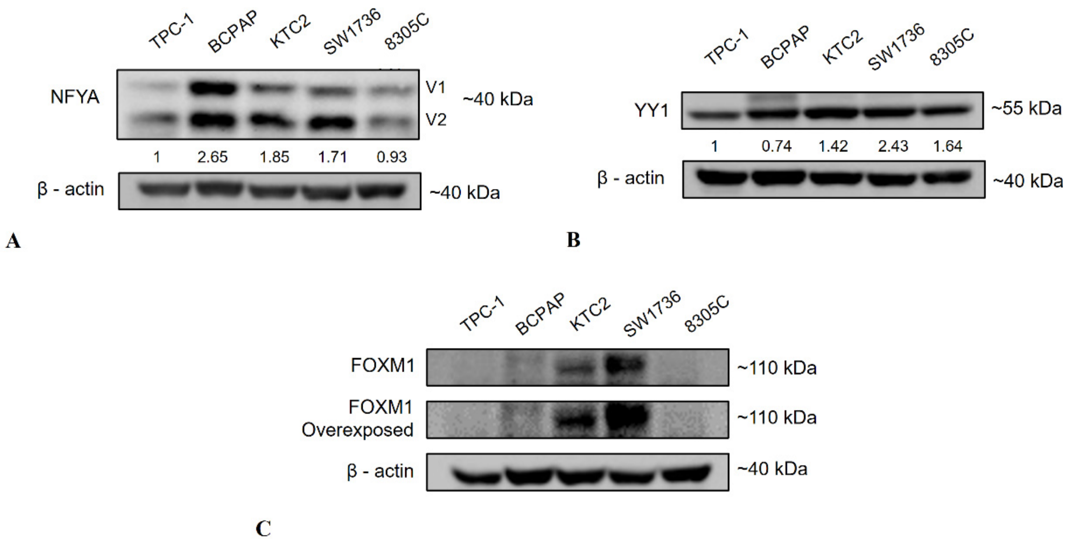

Supplementary Figure S3. Protein expression levels of NFYA (A) YY1 (B) and FOXM1 (C), in PTC (TPC-1 and BCPAP) and ATC (KTC2, SW1736 and 8305C) cell lines by Western-blotting. The protein expression was normalized using β-actin as endogenous control and after band densitometry TFs levels were compared to TPC-1 levels and showed as fold-change rate.

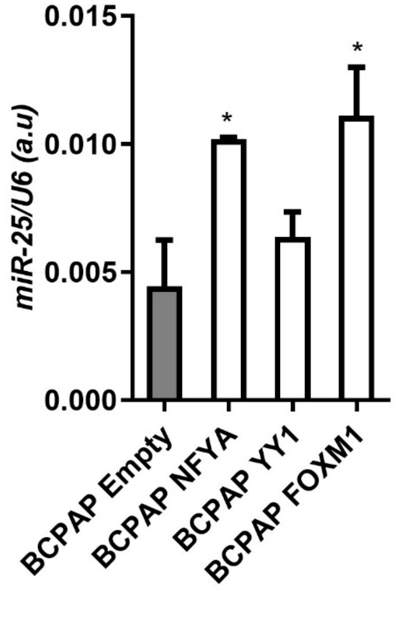

Supplementary Figure S4. *miR-25* expression in response to TFs overexpression. *miR-25* is increased in BCPAP cells overexpressing NFYA and FOXM1. miRNA expression was normalized using *U6* levels. Data represented as mean ± SD (*n* = 3). a.u., arbitrary unit *, $p<0.05$ vs. Empty control.

Supplementary Tables

Supplementary Table S1. Cell culture media used and genetic alterations for each cell line.

Cell line	Culture media	Origin	Main genetic alterations
TPC-1	DMEM 5% FBS	PTC	*RET/PTC1(CCDC6-RET)*
BCPAP	DMEM 10% FBS	PTC	$BRAF^{V600E}$, $TERT^{C228T}$, $TP53^{c.775G>T}$
KTC2	RPMI 5% FBS	ATC	$BRAF^{V600E}$, $TERT^{C228T}$
SW1736	RPMI 10% FBS	ATC	$BRAF^{V600E}$, $TERT^{C228T}$, $TP53^{c.574C>T}$
8305C	RPMI 10% FBS	ATC	$BRAF^{V600E}$, $TERT^{C250T}$, $NRAS^{c.270delT}$

Supplementary Table S2. Primers used for *EZH2* gene promoter fragments cloning and shRNA and TFs over expression plasmid construction.

Primer	Sequence 5' - 3'	Aim	Enzymes
proEZH2-Fw1	gttgttCTCGAGGGTTGAGGTAGCTTGACCCC	cloning fragment E1	XhoI in pGL4-20 MinP plasmid
proEZH2-Rv1	gttgttAGATCTAAACCTGTGAAAGGGCGACA		BglII in pGL4-20 MinP plasmid
proEZH2-Fw2	gttgttCTCGAGACACTGACACGTGCTTAGAACT	cloning fragment E2	XhoI in pGL4-20 MinP plasmid
proEZH2-Rv2	gttgttAGATCTGTGGTAACGGTTTTAACCGCCG		BglII in pGL4-20 MinP plasmid
proEZH2-Fw3	gttgttCTCGAGCCCCCGAGTTTTGAACTGGT	cloning fragment E3	XhoI in pGL4-20 MinP plasmid
proEZH2-Rv3	gttgttAGATCTCCCAATCGCCATCGCTTTTA		BglII in pGL4-20 MinP plasmid
proEZH2-Fw4	gttgttCTCGAGCCCTGTGATTGGACGGGC	cloning fragment E4	XhoI in pGL4-20 MinP plasmid
proEZH2-Rv4	gttgttAGATCTCCGAAGCTCACAGCTCCTTC		BglII in pGL4-20 MinP plasmid
proEZH2-Fw5	gttgttCTCGAGGTCCGCACTCAGACAAAGGA	cloning fragment E5	XhoI in pGL4-20 MinP plasmid
proEZH2-Rv5	gttgttAGATCTAAGTGGCAACTCACTAGGCG		BglII in pGL4-20 MinP plasmid
proEZH2-E3/4Fw	gttgttCTCGAGCCCTGTGATTGGACGGGC	cloning fragment E3/4	XhoI in pGL4-20 MinP plasmid
proEZH2-E3/4Rv	gttgttAGATCTCAAACGCGGCAGCCCAAT		BglII in pGL4-20 MinP plasmid
proEZH2-E3A Fw	gttgttCTCGAGCCCCCGAGTTTTGAACTGGT	cloning fragment E3A	XhoI in pGL4-20 MinP plasmid
proEZH2-E3A Rv	gttgttAGATCTacgcggggaccgggagacat		BglII in pGL4-20 MinP plasmid
proEZH2-E3B Fw	gttgttCTCGAGcctgcacaccgccttcctg	cloning fragment E3B	XhoI in pGL4-20 MinP plasmid
proEZH2-E3B Rv	gttgttAGATctcggaggcgccgagccag		BglII in pGL4-20 MinP plasmid
miR-XhoI-fw	TGAACTCGAGAAGGTATATTGCTGTTGACAGTGAGCG	miR-E cloning	XhoI in sGEN plasmid
miRE-EcoOligo-Rv	GCCTACTGCCTCGGACTTCAAGGGGCTAGAATTCGAGa	miR-E cloning	EcoRl in sGEN plasmid
miRseq5	TGTTTGAATGAGGCTTCAGTAC	miR-E sequencing	
NFYA clon Fw	gttgttCTCGAGatggagcagtatacagcaaa	Overexpression of NFYA	XhoI in pMSCV-puro
NFYA clon Rv	gttgttGAATTCttaggacactcggatgatct	1044bp amplicon	EcoRI in pMSCV-puro
YY1 clon Fw	gttgttCTCGAGatggcctcgggcgacaccct	Overexpression of YY1	XhoI in pMSCV-puro
YY1 clon Rv	gttgttGAATTCcactggttgtttttggcctt	1244bp amplicon	EcoRI in pMSCV-puro
FOXM1 clon Fw	gttgttCTCGAGatgaaaactagcccccgtcg	Overexpression of FOXM1	XhoI in pMSCV-puro
FOXM1 clon Rv	gttgttGAATTCctactgtagctcaggaataa	2256bp amplicon	EcoRI in pMSCV-puro

Supplementary Table S3. Predicted transcription factors that bind to the E3/4 promoter region.

**Putative transcription factors**		
GFI1	ARID3A	E2F
GATA1	NKX6-1	ZEB
NFY	SP1	C/EBPalph a
NFYA	PBX1	FOXM1
NKX2-5	myogenin/ NF-1	RB1
GATA3	KLF4	MITF
YY1	AHR ARNT	NFIC

Supplementary Table S4. Primers used for site-directed deletion analysis.

**Primer**	**Sequence 5' - 3'**	**Site deletion**
Del YY1 Fw	GATTGGGCTGCCGCGTTT	GATGGC
Del YY1 Rv	GCTTTTATTTGGCCCCCC	
Del E2F1 Fw	TCGGGGCGGCGCTTGATTGG	CGTCCCGCCAA
Del E2F1 Rv	CGAGGCGGGCGCCCGTCC	
Del NFYA Fw	GGCGCTTGATTGGGCTGGGGGG	TCCCGCCAATCGGGGC
Del NFYA Rv	CGCGAGGCGGGCGCCCGT	
Del FOXM1 Fw	AAAGCGATGGCGATTGGGCTG	CAAATA
Del FOXM1 Rv	GCCCCCCCAGCCCAATCA	
Del SP1 Fw	TTGATTGGGCTGGGGGGG	GGGGCGGCGC
Del SP1 Rv	GATTGGCGGGACGCGAGG	
Del GATA3 Fw	CTGCCGCGTTTGAG	CGATTGGG
Del GATA3 Rv	CCATCGCTTTTATTTGGC	

Supplementary Table S5. short hairpin (shRNA) guide sequences that aim *FOXM1* gene used for cloning in sGEN plasmid.

**Target**	**Antisense guide sequence**	**Sequence 5'- 3' (97-mer)**
FOXM1 (NM_021953.4)	TTAATAATCTTGATCCCAGCTG (shRNA1)	TGCTGTTGACAGTGAGCGAAGCTGGGATCAAGATTATTAATAGTGAAGCCACAGATGTATTAATAATCTTGATCCCAGCTGTGCCTACTGCCTCGGA
FOXM1 (NM_021953.4)	TATAATTAGAGGATAATTTGGA (shRNA2)	TGCTGTTGACAGTGAGCGCCCAAATTATCCTCTAATTATATAGTGAAGCCACAGATGTATATAATTAGAGGATAATTTGGATGCCTACTGCCTCGGA
FOXM1 (NM_021953.4)	TTACATTTATAATTAGAGGATA (shRNA3)	TGCTGTTGACAGTGAGCGCATCCTCTAATTATAAATGTAATAGTGAAGCCACAGATGTATTACATTTATAATTAGAGGATATGCCTACTGCCTCGGA

Supplementary Table S6. qPCR primers used for gene expression analysis.

Primer	Sequence 5'-3'	Amplicon size	Concentration
E2F1 Fw	ACGTGACGTGTCAGGACCT	146bp	200 nM
E2F1 Rv	GATCGGGCCTTGTTTGCTCTT		
FOXM1 Fw	CCTTCTGGACCATTCACCCC	120bp	200 nM
FOXM1 Rv	TCGGTCGTTTCTGCTGTGAT		
KLF4 Fw	CCCACATGAAGCGACTTCCC	170bp	400 nM
KLF4 Rv	CAGGTCCAGGAGATCGTTGAA		
NFYA Fw	CAGTGGAGGCCAGCTAATCAC	138bp	400 nM
NFYA Rv	CCAGGTGGGACCAACTGTATT		
NKX2-5 Fw	AGCCGAAAAGAAAGAGCTGTG	236bp	200 nM
NKX2-5 Rv	ACCAGATCTTGACCTGCGTG		
YY1 Fw	GTTCAGGGATAACTCGGCCA	157bp	200 nM
YY1 Fw	TTCGAACGTGCACTGAAAGG		
NF1 Fw	TGAAGTGGATCCTACCAGGTTA	183bp	200 nM
NF1 Rv	GCTTTATTCAGTAGGGAGTGGCA		
RPL19 Fw	GGCTGTGATACATGTGGCGA	139bp	400 nM
RPL19 Rv	GGCATAGGTAAGCGGAAGGG		
SP1 Fw	CCACCATGAGCGACCAAGAT	113bp	200 nM
SP1 Rv	TGAAAAGGCACCACCACCAT		
GATA3 Fw	GTCCTGTGCGAACTGTCAGA	226bp	200 nM
GATA3 Rv	CGAGCTGTTCTTGGGGAAGT		
EZH2 Fw	TACTTGTGGAGCCGCTGAC	110bp	200 nM
EZH2 Rv	CTGCCACGTCAGATGGTG		
ZEB1 Fw	GATGACCTGCCAACAGACCA	100 bp	200 nM
ZEB1 Rv	GCCCTTCCTTTCCTGTGTCA		
ZEB2 Fw	AGTGTGCCCAACCATGAGTC	155 bp	200 nM
ZEB2 Rv	TCCTTCATTTCTTCTGGACCATC		

Supplementary Table S7. Antibody and dilution/incubation information used in Western blotting.

Antibody	Concentration	Incubation	Company	Product code
rabbit anti-NFYA	1 : 500	BSA - Overnight 4°C	Invitrogen	PA5-118825
rabbit anti-NFYA	1 : 500	BSA - Overnight 4°C/ 2h at room temperature	Invitrogen	#MA5-36198
rabbit anti-YY1	1 : 500	BSA - Overnight 4°C	Invitrogen	712089
rabbit anti-FOXM1	1 : 1000	BSA - Overnight 4°C	Cell Signaling	#5436
rabbit anti-ERK-1	1 : 1000	Milk - 2 h at room temperature	Santa Cruz	#SC - 94
mouse anti-p-ERK	1 : 1000	BSA - Overnight 4°C	Santa Cruz	#SC 7383
rabbit anti-EZH2	1 : 1000	Milk - 2 h at room temperature	Cell Signaling	#5246
mouse anti-β-actin	1 : 1000	Milk - 2 h at room temperature	Santa Cruz	#SC 47778

TTBS: TBS containing 0.05% Tween 20

Milk: 5% skim milk in TTBS

BSA: 3% BSA in TTBS

Supplementary Table S8: qPCR primers used for CUT & RUN analysis.

Primer	Sequence 5'-3'	Amplicon size	Concentration
**E3/4 CUT & RUN Fw**	CTCGCGTCCCGCCAATC	70 bp	200 nM
**E3/4 CUT & RUN Rv**	CCCAATCGCCATCGCTTTTA		
